# Neuropsychological Symptoms and Quality of Life during the COVID-19 Pandemic in Children: A Survey in a Pediatric Population in the Abruzzo Region, Italy

**DOI:** 10.3390/children11050532

**Published:** 2024-04-28

**Authors:** Chiara Marcotullio, Marina Attanasi, Annamaria Porreca, Paola Di Filippo, Sara Matricardi, Annamaria Venanzi, Marco Schiavo, Antonio Paone, Nadia Rossi, Francesco Chiarelli, Giovanni Prezioso

**Affiliations:** 1 Department of Pediatrics, University of Chieti-Pescara, 66100 Chieti, Italy; chiara.marcotullio@studenti.unich.it (C.M.); marina.attanasi@unich.it (M.A.); difilippopaola@libero.it (P.D.F.); sara.matricardi@unich.it (S.M.); anna.ven@outlook.it (A.V.); marco.schiavo@studenti.unich.it (M.S.); nadia.rossi@asl2abruzzo.it (N.R.); chiarelli@unich.it (F.C.); 2 Laboratory of Biostatistics, Department of Medical, Oral and Biotechnological Sciences, “G. d’Annunzio” University of Chieti-Pescara, 66100 Chieti, Italy; annamaria.porreca@unich.it; 3 Department of Neuroscience, Imaging and Clinical Science, University of Chieti-Pescara, 66100 Chieti, Italy; antonio.paone001@studenti.unich.it

**Keywords:** COVID-19, long COVID, neuropsychology, emotional well-being, social habits, quality of life

## Abstract

Background: The SARS-CoV-2 pandemic has significantly affected the pediatric population. Long-term sequelae (Long COVID-19) may particularly involve the central nervous system, with possible effects on psychological well-being and quality of life (QoL), aspects that were already influenced by the restrictive measures and general social impact of the pandemic. Methods: We conducted a cross-sectional survey that aims at investigating the neuropsychological effects and the QoL impairment of SARS-CoV-2 on a cohort of children and adolescents in the Abruzzo region (Italy). A questionnaire was submitted to caregivers with the help of the PEDIATOTEM platform. A control group of healthy subjects was also included to distinguish between the effects of infection from the general influence of the pandemic. Results: A total of 569 subjects responded: 396 COVID-19 patients (99 of whom had Long COVID-19) and 111 controls. After the pandemic, when compared with the COVID-19 group, the controls reported significantly increased appetite, sleeping habits, and time spent remotely with friends and a reduction in physical activity and time spent in person with friends. A significant higher rate of controls asked for psychological/medical support for emotional problems. On the other hand, the Long COVID-19 group showed more fatigue and emotional instability with respect to non-Long-COVID-19 subjects. No differences in QoL results (EuroQOL) were found between the COVID-19 patients and controls, while the Long-COVID-19 subgroup showed significantly higher rates of pain/discomfort and mood instability, as confirmed by the analysis of variation of responses from the pre-COVID-19 to the post-COVID-19 period. Conclusions: Among COVID-19 patients, neuropsychological and QoL impairment was more evident in the Long COVID-19 subgroup, although emotional and relational issues were also reported by uninfected patients, with a growing request for specialist support as a possible consequence of social restriction.

## 1. Introduction

The Coronavirus disease 2019 (COVID-19) pandemic, caused by Severe Acute Respiratory Syndrome Coronavirus 2 (SARS-CoV-2), continues to spread, causing significant mortality and morbidity worldwide [1,2,3].

In children, acute SARS-CoV-2 infection is often mildly symptomatic or asymptomatic, and more serious and potentially life-threatening complications are rare [3,4].

However, despite the low risk of severe acute COVID-19 in the pediatric population, it has been widely demonstrated that SARS-CoV-2 infection can lead to the onset of long-term multisystem sequelae, currently referred to as Long COVID-19 [1,5,6].

The World Health Organization (WHO) proposed a consensus defining Long COVID-19 as a condition typically occurring three months after the onset of a probable or confirmed SARS-CoV-2 infection, with symptoms lasting at least two months and unable to be explained by an alternative diagnosis [7].

Most published epidemiological studies and reviews focus primarily on Long COVID-19 among adult populations, while data regarding the long-term effects of COVID-19 in the pediatric population are still limited [8].

As in adults, studies conducted on the pediatric population identified having a female gender, severe forms of COVID-19, obesity, allergic diseases, and other comorbidities as risk factors for the development of Long COVID-19 [5,9].

In a recent meta-analysis, combining data from 21 studies with over 80 thousand children and adolescents, Lopez et al. [5] found that the prevalence of Long COVID-19 among the pediatric population was 25.24%. The most common symptoms were mood symptoms (16.50%), fatigue (9.66%), sleep disorders (8.42%), headaches (7.84%), and respiratory symptoms (7.62%) [5].

Other studies confirmed that most of the Long COVID-19 symptoms are neurological and neuropsychiatric, such as headaches, altered cognition (‘brain fog’), joint and muscle pain, anxiety, depression, and sleep disorders [10,11,12].

The prevalence of neuropsychiatric symptoms has led to the question of whether they were related to a direct action of the SARS-CoV-2 infection or determined by the stressors of the pandemic.

Several studies suggest that neurological and neuropsychiatric symptoms may differ in the underlying pathophysiology. SARS-CoV-2 infection seems to have a key role in the development of neurological symptoms, while social isolation and pandemic-related restrictions seem to play a relevant role in neuropsychiatric manifestations, such as mood problems [6,13,14,15].

Understanding the problem of neuropsychiatric manifestations of Long COVID-19 is essential to allow for the timely recognition of affected children and to implement adequate support through pediatric-healthcare resources.

However, the absence of a control group in most studies regarding the neuropsychiatric manifestations of Long COVID-19 in children and adolescents makes it difficult to distinguish between symptoms directly attributable to the infection and those related to the pandemic.

For this reason, in our study, we aimed to compare, in a pediatric population from Abruzzo (Italy), the neuropsychological effects and the impact on quality of life and social habits among children with a recent SARS-CoV-2 infection and a group of healthy controls, to distinguish the direct effects of COVID-19 from pandemic-related symptoms.

## 2. Materials and Methods

A cross-sectional survey was addressed to patients aged 6–18 with a previously documented SARS-CoV-2 infection and a group of healthy controls of the same age without a previous COVID-19 diagnosis. Data were collected in Abruzzo, an Italian region, from September 2022 to January 2023.

The survey was developed using the “International Severe Acute Respiratory and Emerging Infection Consortium” (ISARIC) Global Paediatric Follow-up questionnaire of Oxford University [16], of which we promptly received the Italian version 1.4 (https://isaric.org/.../paediatric-follow-up, accessed on 6 February 2022). The ISARIC survey has been validated and has already been used by other research groups on a population of adult and pediatric COVID-19 patients [17].

This questionnaire investigates the prevalence and risk factors of post-COVID-19 conditions in children. It is subdivided into different sections concerning patients’ characteristics and COVID-19 infection, from the acute phase to the persistent symptoms, analyzing physical and mental health. Comorbidities, vaccination status, and psychological and relational well-being data were also collected (Table 1).

The questionnaire also included the quality-of-life test of the EuroQoL group association, an international network of multidisciplinary researchers, for whom a regular license to use the EQ-5D-Y-3L version for non-commercial projects was obtained (© 2024 EuroQoL Research Foundation). The EuroQoL test analyzes five dimensions (i.e., mobility, looking after myself, doing my usual activities, feeling pain/discomfort, and feeling worried/sad/unhappy) and a visual analogue scale, which examines the general health condition [18]. Each dimension had three possible responses, indicating the presence of problems and their intensity level. Caregivers were asked to respond to the perceptions of these five dimensions before and after the COVID-19 infection (or pandemic, for the control group) (Table 2). The visual analogue scale was not included in our analysis for practical reasons, since it was by phone call that we administered the questionnaire. To compare the psychological wellness and quality of life of COVID-19 patients with a group of subjects without a history of SARS-CoV-2 infection, the questionnaire was properly modified for the control group.

For the recruitment, local primary care pediatricians (PCPs) were enrolled in a phase lasting 3 weeks with the support of PEDIATOTEM, an innovative multi-media system designed to create a network between pediatricians and parents to monitor children’s health, promote communication, and contribute to medical research (https://www.pediatotem.it/PediaTotem_sito/index.html, accessed on 20 November 2023). PCPs were contacted with a notification and a one-week reminder for non-responders. After obtaining PCPs’ consent, responders sent their patients an electronic version of the questionnaire via PEDIATOTEM. The survey included a cover letter and an informed consent that parents had to accept before participation.

The LVIIIER agency contributed to this study free of charge. The agency collected and returned the anonymous data (names were visible only to their PCP). An alpha-numerical code was used to identify each patient.

The study was conducted in accordance with the Declaration of Helsinki and received approval from the Regional Ethics Committee of Abruzzo (C.Et.R.A.).

Three authors (MC, MS, and AV) contacted the parents by telephone twice to administer the EuroQoL test.

Within the COVID group, the Long COVID-19 subgroup was further identified using the WHO definition as a temporal criterion (i.e., persistent symptoms that lasted at least four weeks after the acute COVID-19 infection) [19].

Patients were divided into two age-range subgroups: 6–12 (group 1) and 13–18 (group 2). From the collected data, the first study, aimed at investigating symptoms of acute COVID-19 and Long COVID-19 with its predictive factors, was developed and published [20,21].

### Statistical Analysis

The data were analyzed using descriptive statistics appropriate for the nature of the variables. Continuous variables were presented with means ± standard deviations. Categorical data were presented as absolute frequencies (n) and percentages (%). Pearson’s Chi-squared test was used to investigate the associations between categorical variables. For continuous variables, differences between groups were assessed using the Student’s *t*-test. The normality assumption was assessed using the Shapiro–Wilk test. All *p*-values were two-tailed, and a *p*-value of ≤0.05 indicated a statistically significant relationship.

All statistical analyses were performed using the R statistical environment (version 4.3 R Foundation for Statistical Computing, Vienna, Austria) [22].

## 3. Results

### 3.1. General Characteristics of the Study Population

The questionnaire was sent to 4823 patients, followed by 10 PCPs, and 600 (12.4%) responded. Of them, 31 were excluded from the analysis because of incomplete data. Thus, a final set of 569 patients was evaluated (396 COVID-19 patients and 111 controls). For further details and the flow chart of the study, please refer to Schiavo et al. [20].

In the COVID group, 194 patients (49%) were males and 202 were females (51%), with a mean age of 9.32 ± 2.55 years, and with 87.9% belonging to the 6-to-12-years age range; 48 (12.1%) declared an ethnicity other than Caucasian. At least one dose of the COVID-19 vaccine was administered to 138 (55.9%) patients.

The control group was represented by 61 (55.0%) males and 50 females (45.0%) with a mean age of 9.41 ± 2.74 years; 75 (91.5%). Most patients were Caucasian, and seven (6.3%) came from other ethnic groups. Eighty-one (72.9%) were vaccinated for COVID-19 (Table 3).

Within the COVID-19 group, 99 patients (25%) fulfilled the Italian National Institute of Health criteria for Long COVID-19 [23]. Among them, 48 (48.5%) patients were males, and 51 (51.5%) were females, with a mean age of 9.0 ± 2.68 years and a prevalence of children in the age range of 6 to 12 years (84.8%). Seven (7.0%) patients were not Caucasian; 60 (61.9%) were vaccinated for COVID-19. The following persistent neurological symptoms were reported: tiredness (23.2%), a need for more rest (20.2%), concentration problems (15.2%), sleepiness (16.2%), decreased muscle strength (14.1%), a lack of energy (13.1%), weakness (11.1%), difficulty starting activities (11.1%), memory (9.09%), difficulty with recalling words (9.09%), and lapses/mistakes in speech (8.08%).

### 3.2. Social Habits and Emotional Well-Being

**Figure 1:**
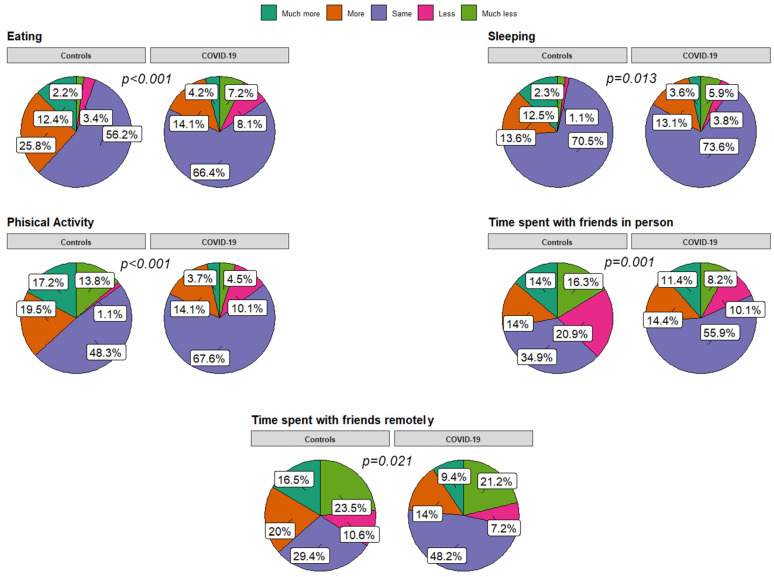
Pie charts for responses related to social relationships and emotional well-being after COVID-19 infection or pandemic outbreak (for uninfected controls) compared to the time before infection/pandemic.

**Figure 2:**
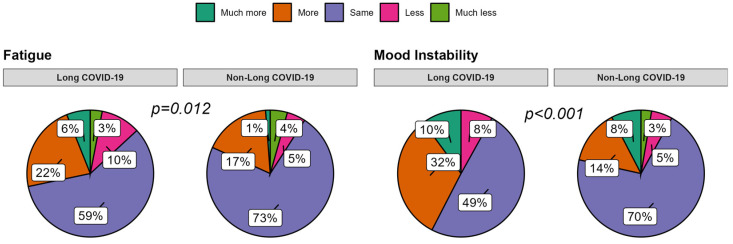
Pie charts for responses related to social relationships and emotional well-being after COVID-19 infection compared to the time before infection in the Long COVID-19 group versus the Non-Long COVID-19 group.

Concerning emotional well-being and social habits, significant differences were found in the comparison between the COVID group and the control group in terms of eating (*p* < 0.001), sleeping habits (*p* = 0.013), and physical activity (*p* < 0.001). In particular, the control group perceived an increased appetite, sleeping habits, and physical activity (PA) after the pandemic onset compared to COVID patients. They also noticed a reduction in time spent with their friends in person (*p* = 0.001), as opposed to an increased time spent remotely (*p* = 0.021) (Figure 1 and Figure 2). No differences between the groups were found for the use of electronic devices, and time spent outdoors increased in both groups. Similarly, no significant differences existed in school attendance, fatigue perception, connection, and emotional lability.

When asked about the need for psychological/medical support for emotional/relational issues, the controls declared a rise in requests compared to the COVID group (*p* = 0.018). In detail, 13 controls (11.7%) versus 23 COVID-19 patients (5.8%) sought help for their psychological problems after the pandemic outbreak (*p* = 0.02). Overall, 11 patients (30.5%) sought help from their PCP, 16 (4.4%) from psychologists, and 9 (25.1%) from other figures.

By analyzing the Long COVID-19-subgroup answers, significant differences compared to the COVID-19 patients without Long COVID-19 (No-Long COVID-19 group) were found for fatigue (*p* = 0.012) and emotions (*p* < 0.001), with the first group perceiving more fatigue and emotional instability. No further differences resulted from the remaining fields explored.

After correcting for age and gender, the above-mentioned differences persisted.

### 3.3. Quality of Life

From the EuroQol test results, no significant variation was found for the five dimensions (mobility, self-care, usual activities, pain/discomfort, and feeling worried/sad/unhappy) between the responses related to the pre-COVID/pandemic and post-COVID-19/pandemic conditions in both the COVID-19 group and the controls. Within the COVID-19 group, a mild increase in pain/discomfort (3.6% of responders) and mood disorders (5.6% of responders) was observed after COVID-19 infection. The controls declared minimal changes, mainly for pain/discomfort (4%), when compared to their pre- and post-pandemic condition. Feeling worried/sad/unhappy was the only dimension that obtained “level 3” answers (i.e., many problems) in both the control and COVID-19 groups, with a higher rate of difficulties reported before (12% vs. 10.7%) and after (13.4% vs. 16.7%) the pandemic outbreak.

**Figure 3:**
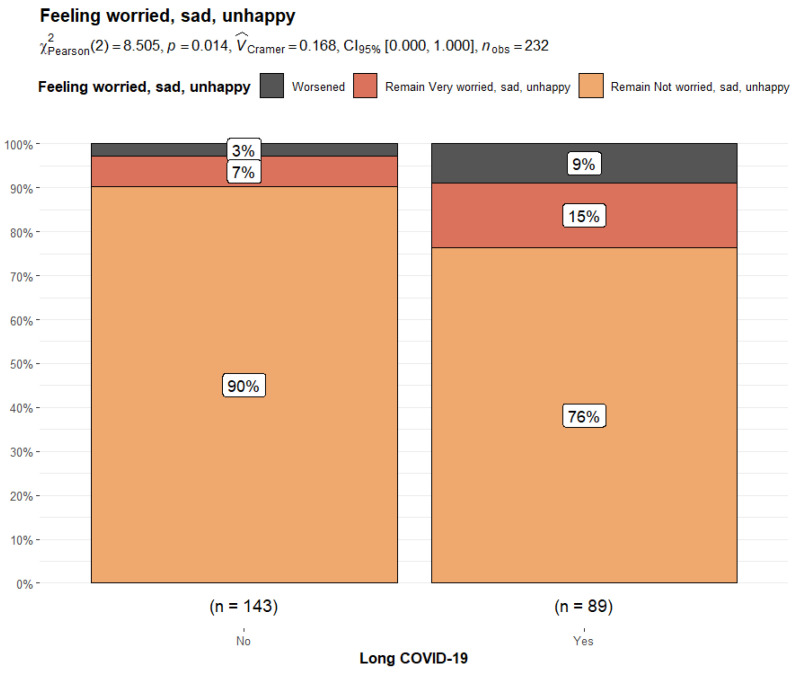
Bar plot and Chi-squared test for changes in responses to the EuroQoL dimension “feeling worried/sad/unhappy” from the pre-COVID-19 era to the post-COVID-19 condition in Non-Long COVID-19 patients versus Long COVID-19 patients.

**Figure 4:**
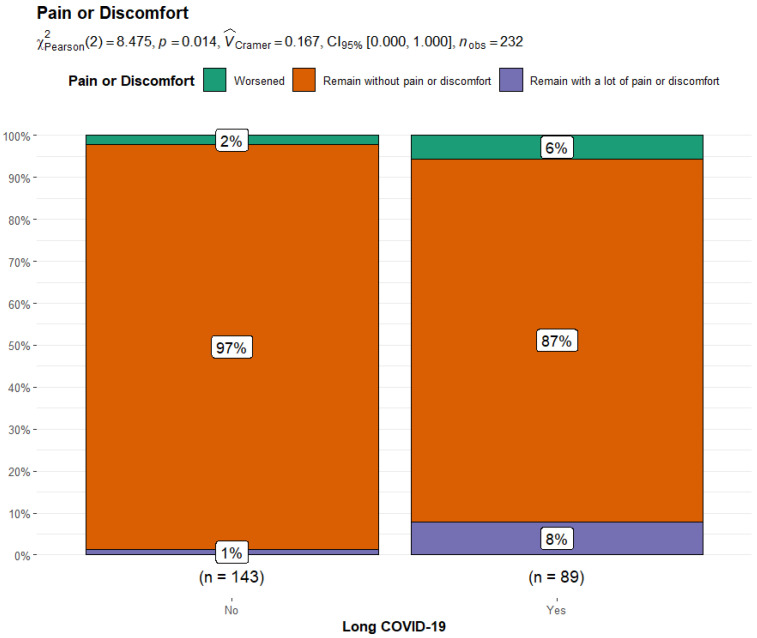
Bar plot and Chi-squared test for changes in responses to the EuroQoL dimension “pain/discomfort” from the pre-COVID-19 era to the post-COVID-19 condition in Non-Long COVID-19 patients versus Long COVID-19 patients.

By comparing the Long COVID-19 and Non-Long COVID-19 groups, the former reported significant differences in their perception of pain/discomfort (14.4% vs. 3.4%, *p* = 0.004) and feeling worried/sad/unhappy (24.2% vs. 9.59%, *p* = 0.005) after COVID-19 infection. They also reported a significantly higher rate of pain/discomfort (7.9% vs. 1.4%, *p* = 0.029) and mood disorders (14.6% vs. 6.9%; *p* = 0.049) before COVID-19 infection compared to Non-Long COVID-19 patients. When the variation in responses related to the pre-COVID-19 and post-COVID-19 phases was compared, it was noticed that 6% of Long COVID-19 patients experienced a worsening in pain/discomfort perception compared to the 2% of Non-Long COVID-19 patients (*p* = 0.014). Similarly, 9% of subjects with Long COVID reported a worsening in mood compared to 3% of Non-Long COVID-19 patients (*p* = 0.014) (Figure 3 and Figure 4).

## 4. Discussion

The impact of SARS-CoV-2 on social habits was evident in the first phase of the pandemic, when restrictive measures were established by governments aimed at containing virus diffusion. Quarantine, social distancing, physical restrictions, school closures, indoor activities, and sports limitations have certainly provoked abrupt effects on children’s psychosocial and mental health [24,25,26]. Evidence from the literature and data from real-world medicine support the hypothesis that emotional and behavioral symptoms persisted even after the first phase of the pandemic [27,28,29]. A remarkable role was played by the virus, which has been shown to interact with the central nervous system directly and indirectly, bringing on acute and persistent symptoms that may impair a child’s quality of life [11,12,30,31,32,33]. The symptoms of the nervous system can be divided into specific and non-specific symptoms [10]. Data from a US registry of 1965 hospitalized children found that 22% complained of neurological symptoms; of these, 12% had life-threatening conditions like encephalopathy, stroke, CNS infections, or demyelinating diseases [34]. A multicenter study from the Clinical Characterization Group of the ISARIC Consortium tested 161 239 patients (of whom 2972 were children) who were admitted to a hospital for COVID-19 and found that the most common acute neurological symptoms in children were fatigue, myalgia, seizure, anosmia, and dysgeusia [35]. Another common acute symptom described is headaches [5,36], which are more frequent in young people and patients with a previous diagnosis of primary headaches [37]. Indeed, in our previous publication on this survey, we found that 49.5% of COVID-19 patients complained of headaches in the acute infection, without a gender or age prevalence [20].

As for adults, CNS dysfunction seems to persist for a long time in a significant portion of infected pediatric patients, resulting in so-called Long COVID-19 [1,5,6,38,39]. Headache, fatigue, ‘brain fog’ and memory impairment, anosmia or ageusia, and dizziness are the most frequent symptoms associated with Long COVID-19, with a highly variable prevalence among the studies [1,5,6,40].

Neuropsychiatric symptoms are also described in patients with Long COVID-19, especially mood symptoms and anxiety, affecting their daily functioning and well-being [6,8,41]. The direct effects of the virus are not the sole contributors, as pandemic-related psychosocial factors have also influenced this group of patients in an unclear manner.

Moreover, the insufficient number of large prospective studies on pediatric populations, along with the great variability in patients’ selection, data collection, and investigation methods, brought about considerably different results among countries and studies [10,42]. There is a growing demand for controlled studies in the literature to help distinguish between possible SARS-CoV-2 effects and pure psychosocial factors of the pandemic.

### 4.1. Social Habits and Emotional Well-Being

In line with these needs, our study involved a relatively large sample of the local pediatric population. It included a control group with a homogeneous gender distribution and a prevalence of children below 13 years of age.

Our results revealed that the COVID-19 and control groups perceived the impact of the SARS-CoV-2 pandemic on their social habits and emotional well-being differently, at least to some degree.

More in detail, when comparing eating habits, an increase in appetite was more evident in the control group (38.2% vs. 18.3%), without gender and age differences. The previous literature has signaled a worsening in eating habits and an increased appetite during the lockdown in different samples of European children [43,44,45,46,47], especially in females and in at-risk populations (like obese children). Among the possible explanations, the first role of outdoor-activity restriction with consequential sedentary behaviors was certainly substantial. A parental psychosocial factor has also been demonstrated by Varghese et al. [48]: they found that parenting stress was positively correlated to lower mindful feeding and children’s obesogenic eating behaviors [48]. A Dutch study documented a similar association with adolescent-parenting practices [49]. Less is known about how these habits evolved after the first years of the pandemic. A systematic review conducted in 2022 by an Iranian group concluded that eating-habit changes were controversial in children and adolescents, with both a decrease and rise in positive and negative behaviors. Our study lacks a prospective design; however, it seems to suggest a prevalence of increased appetite perception in non-exposed subjects to COVID-19.

On the other hand, more than 60% of caregivers of COVID-19 patients did not find remarkable changes in appetite to their pre-COVID-19 state. However, a higher rate of children complained of reduced appetite than the control group (15.3% vs. 5.6%). Analogous data was obtained from the Long COVID-19 group. A direct effect of SARS-CoV-2 on eating habits has not been clarified. However, some authors suggested that the transient alteration in taste and smell chemosensory receptors might play a role [50] in altered food intake. This may resolve quickly after acute infection, except for cases with persistent symptoms, showing a higher risk of eating-disorder development [51]. In the last two years, due to the fast emergence of new variants showing reduced effects on chemosensory dysfunction [52], COVID-19’s role in eating habits may be less evident, as shown in our survey.

Of note, the control group also reported a significantly higher need for sleep than the COVID-19 group (level 5: 12.5% vs. 3.6%), without differences in age and gender. Conversely, no differences were found between the Non-Long COVID-19 and Long COVID-19 groups. However, when asked about last month’s symptoms, caregivers of Long COVID-19 children reported a significantly increased rate of tiredness, the need for rest, and somnolence. Previous studies described a general trend towards increased sleep disturbances that was more evident during the initial lockdown when daily routines were dramatically interrupted. In that phase, a sleep-quality and -duration impairment was found, with evidence of bedtime and waketime delay, a longer duration of sleep, parasomnias, and daily nap reduction [53,54]. The reduction in outdoor activities on one side and the increased exposure to light from screens and stress on the other may have been crucial [55,56,57]. In other studies, no overall change in sleep duration was found, despite a delay in bedtime and waketime, with a likely protective effect of parental education [58]. By contrast, some authors highlighted improved sleep quality in adolescents and young adults due to the more flexible virtual learning programs and remote working [59]. Less is known about the effects on sleep in the latest pandemic period. Our results suggest that sleep-time disorders may recur in a subgroup of patients, which needs to be better defined in future studies, while, in Long COVID-19 cases, sleep-quality problems may be more relevant than total sleep time.

Concerning PA, the answers provided by the caregivers revealed that about 36% of the Non-COVID-19 group were more active than in the pre-pandemic era, as opposed to 17% of the COVID-19 group (rising to 21% in the Long COVID-19 cases). Similarly, fatigue perception was not increased among the controls, while 21% of COVID-19 patients and 28% of Long COVID-19 patients reported increased fatigue, without gender or age effects. A meta-analysis performed on 16 studies extended to pediatric patients found a predominance in PA decline irrespective of gender (eight studies), with only two studies clearly showing PA levels comparable to ours [60]. Among contributing factors, a male gender, online physical education classes, the use of digital platforms, access to parks, prior healthy habits, and encouraging family contexts were found [61]. The remaining studies reported mixed results, probably because of several additional influencing factors. These include different social restrictions and education policies between countries, geo-social and cultural differences, socio-demographic factors (i.e., population density), and heterogeneity between studies and data collection. Other possible negative factors reported in a similar study ranged from the risk of COVID-19 exposure, insufficient instruction, inadequate access to sports, and poor local climatic conditions [62]. Positive factors included family facilitation, closeness to outdoor spaces, and perception of mental-health benefits [62]. With the above-mentioned limitations, the response of our local population to the last phase of the pandemic appears generally positive, with a predominance of no substantial perception of PA changes in all groups and even an increase in PA in Non-COVID-19 subjects. Of note, PA in patients with Long COVID-19 did not seem to be significantly modified by the disease, despite the increased fatigue reported by almost one-third of the subjects, and the symptoms were reported to be persistent after acute infection. Persistent fatigue in Long COVID-19 has already been described in adults and children, with a pediatric prevalence ranging from 10.8% to 71% [17,63,64,65]. A possible concurrent effect of neuroinflammation, psychosocial triggers, and genetic predisposing factors have been hypothesized [66], with a direct impairment on neural metabolism demonstrated by Sollini et al. [67] through F-FDG PET alterations in the right parahippocampal gyrus, brain stem, and thalamus [67]. However, it should be noted that the definition of fatigue is highly subjective and difficult to measure to explain the large variability among studies [6]. For this reason, a study group from Western countries has proposed a definition for post-COVID-19 fatigue, considering the persistence of disabling symptoms for three months or more after SARS-CoV-2 infection. Using a uniform definition in future studies will contribute to a better understanding of the impact of COVID-19.

Interestingly, the perception of time spent with friends in person and remotely seems more affected in the control group than in the COVID-19 and Long COVID-19 groups. A possible psychosocial explanation may be the tendency of unexposed subjects to maintain adherence to mitigation measures after the lockdown phase in contrast to patients who got COVID-19, as indicated in other studies [68].

As expected, the time spent on electronic devices for educational and non-educational purposes was perceived as increased in both COVID-19 and No COVID-19 groups, with frequencies ranging from 34.6% to 46.4%. Again, a mild but non-significant predominance of Non-COVID-19 patients was noted.

Internet overexposure may be explained by the need to interact with peers, find alternatives for self-realization, and, more generally, occupy themselves in a period of limited interactions. In this regard, social media has become increasingly influential in sharing content and connecting people worldwide in real-time. One of the issues parents should be aware of is the risk of oversharing, which can lead to internet addiction, information overloading, personal data exposure, attention seeking, recognition problems, anxiety problems, or cyberbullying.

Thus, families and teachers should carry out appropriate child education for the safe use of digital devices, in parallel to the close monitoring of children’s activities and unusual/abnormal behaviors [69].

Regarding school/work attendance, most COVID-19 and control patients did not report changes compared to the pre-COVID-19 era; this data may be influenced by the time the survey was performed, since restriction measures were largely reduced in Italy in late 2022.

Results about emotional and behavioral modifications deserve particular attention. Despite moderate impairment in both COVID-19 and control groups without significant differences, when Long COVID-19 patients were evaluated, 42% and 31% of them, respectively, showed an increase in emotional and behavioral instability, regardless of age and gender and with a significant difference in comparison to Non-Long COVID-19 patients. A meta-analysis performed by Lopez-Leon et al. [5] showed that mood symptoms were in first place among the most common manifestations of Long COVID-19 (16.5%), followed by fatigue, sleep disorders, and headaches [5]. In addition to the expected role of long-term COVID-19’s neurological symptoms, discussed later, it has been hypothesized that the presence of prior mental-health difficulties and multiple symptoms in the acute phase of the disease could be risk factors for symptom persistence [70]. Thus, our results may reflect a predisposition of the Long COVID-19 group to psychological disorders.

Interestingly, the patients unexposed to SARS-CoV-2 were the ones who asked more for support for the above-mentioned issues, regardless of a pre-pandemic psychological/psychiatric consultation. These results support previous data, showing a trend of increased mental-health problems in the general population after the SARS-CoV-2 outbreak, with a peak in adolescence [26,71,72,73]. Patients with pre-existing psychological distress or psychiatric illness have a high risk of worsening their symptoms during a pandemic. Still, our study shows that previously healthy subjects have also been affected in our area. A rise in anxiety and depression has already been documented, with a trend related to the different phases of the pandemic (lockdowns and re-openings) [27]. Some authors also highlighted a correlation between behavioral changes in children during quarantine and parental sleep problems and stress, with the protective factor of caregivers’ coping strategies for younger children [74]; parental economic stress and worries about children’s performances were also proved to be related to emotional/behavioral issues [75].

Therefore, further multicenter studies should focus on the possible long-term effects of pandemics on pediatric mental health, with special attention given to caregivers’ skills and predisposing factors. Moreover, local authorities must prioritize and implement mental-support programs in schools and public health services to identify the most vulnerable subjects. Several social-intervention strategies have the potential to prevent and support patients/families in danger: from coping strategies to emotional support, and from family mediation to crisis intervention. Social work remains essential to connect caregivers, schools, psychological services, and local institutions. It also guarantees social rehabilitation when required to remove conditions that threaten a child’s normal biological, social, or psychological development [76].

### 4.2. Quality of Life

An impairment of emotional well-being may affect a child’s overall QoL and functioning.

When we tested COVID-19 patients and controls for QoL parameters, our results did not reveal any statistically significant difference in responses regarding mobility, self-care, usual activities, or mood instability before or after the pandemic. This lack of discernible divergence between the groups suggests a certain level of uniformity in the perceived quality of life, regardless of whether the children contracted COVID-19. However, an increased perception of emotional instability can be inferred in a minority of patients from both groups after the pandemic onset.

Only for the section on pain-related responses, a greater proportion of children and adolescents within the COVID-19 group reported heightened pain perception and discomfort compared to the control group. Although this discrepancy did not reach statistical significance (7.8% vs. 1.9%, *p*-value 0.063), it is noteworthy as it suggests a trend toward increased pain perception among COVID-19-affected individuals. However, upon further examination within the COVID-19 group, a significant disparity emerged between the Long COVID-19 and Non-Long COVID-19 groups. Specifically, 14.4% of Long COVID patients versus 3.4% of those without Long COVID reported pain symptoms after infection, and these results were statistically significant (*p* = 0.004). Of note, a significantly higher rate of Long COVID-19 patients complained of pain before and after the SARS-CoV-2 infection (8% vs. 1% of Non-Long COVID-19).

Several studies have highlighted the occurrence of pain as a prominent symptom in individuals who have previously been infected by SARS-CoV-2 [77,78,79]. While the prevalence of pain symptoms has varied significantly across these studies due to their subjective nature and the self-reporting of symptoms, it is important to note that pain, whether general or specific, has been frequently reported as both a short- and long-term consequence of COVID-19 infection [36,80,81].

In a meta-analysis encompassing 40 studies involving 12,424 children and adolescents, Zheng et al. [8] revealed a substantial prevalence of various pain symptoms among children grappling with Long COVID-19 [8]. The analysis reported significant rates of headaches [15.88% (95% CI 6.85–27.57)], abdominal pain [12.42% (95% CI 2.94–26.81)], muscle pain [11.42% (95% CI 3.45–22.96)], chest pain [5.88% (95% CI 1.27–13.15)], and joint pain in adolescents and children with previous SARS-CoV-2 infections [2.74% (95% CI 0.36–6.74)] [8].

The mechanisms underlying the development of short- and long-term pain manifestations post-COVID-19 remain incompletely understood. Possible contributors may include direct viral effects (such as persistent viremia), hyperinflammatory or autoimmune responses, and neurotropism [10,82,83,84].

Furthermore, the compounding stressors experienced during the pandemic could significantly influence pain perception in children and adolescents. Stress and anxiety, prevalent among individuals of various age groups during the pandemic, have been recognized as significant factors exacerbating the experience and sensitivity to pain [84]. Numerous studies have shed light on the profound effects of the pandemic on pediatric populations, highlighting the rise in pain-related complaints and heightened stress levels [26,85,86].

According to a study by Jones et al. [26], the pandemic-induced disruptions in daily routines, school closures, social isolation, and increased exposure to distressing news have elevated stress levels among children and adolescents [26]. These stressors have been linked to amplified reports of headaches, abdominal pain, and musculoskeletal discomfort in pediatric populations [26].

The psychological impact of prolonged stress has resulted in a heightened sensitivity to pain among pediatric individuals, potentially exacerbating their discomfort and reducing their pain threshold [80].

In our study, the pre-existence of a higher perception of pain within the Long COVID-19 group before infection may reveal a predisposing factor of these subjects to pain or stress, which should be thoroughly investigated in future studies.

While COVID-19 infection and the pandemic may exacerbate pain perception, there may be pre-existing emotional vulnerabilities that play a role in the experience of pain in children and adolescents. For example, prolonged periods of isolation and fears of contagion have had more problematic and long-lasting effects on some age groups, such as adolescence, which is already marked by significant challenges and physiological changes. Furthermore, the social context, economic difficulties, and family history of neuropsychiatric pathologies may have been an aspect on which the pandemic-related stress acted [24,87,88].

Regarding mood impairment, our study revealed significant statistical differences in responses related to concern and depression. The parents of both COVID-19 groups observed an increase in their children’s levels of worry and anxiety, with a significantly higher rate in the Long COVID-19 group compared to patients without Long COVID-19. Of note, 14.6% of patients with Long COVID-19 complained of mood impairment before SARS-CoV-2 infection, as opposed to the 6.9% of Non-Long COVID-19 subjects. A relevant rate of patients with worry and anxiety was also signaled among controls (12%) before the pandemic.

It can be speculated that the pre-existing difference in mood rating within the COVID-19 group could have played a role in the perception of persistent symptoms after acute infection whilst considering the limits of a small sample and the possible influence of other undetected variables.

Moreover, our findings align with numerous studies conducted throughout the COVID-19 pandemic, emphasizing both the negative impact on mental health due to pandemic-related circumstances (social isolation, worries about personal and family health, financial challenges, and uncertainties regarding the future) and the triggering role of prolonged COVID-19 symptoms on worries and anxiety [26,60,71,89,90,91,92].

It is crucial to underscore that the connection between the viral threat, imposed restrictions, and the rise in mental-health issues has been evident across various contexts and populations, including pediatric groups.

Our study also underscores the need for a comprehensive understanding of young individuals’ psychological and emotional well-being, particularly in relation to their experience of physical symptoms. Given the remarkable rate of controls with mood impairment looking for psychological support during pandemics, it is mandatory for clinicians, teachers, and all other relevant professionals who deal with children to focus on their mental-health care.

Finding new strategies, such as providing mental-health support, facilitating access to telemedicine for pain-management consultations, and re-establishing safe social connections, is essential for implementing targeted interventions to alleviate stress in children and adolescents during and after pandemics.

### 4.3. Strengths and Limitations

In our opinion, using medical-messaging software for online surveys is one of the innovative aspects of this study and helped us reach a sufficiently representative sample of the local pediatric population, saving time and money. The integration of digital technologies is a trend that started in the last few years and dramatically exploded in the pandemic era, allowing us to overcome the barriers of social distancing and restrictions [93], not without regulatory issues that institutional organizations have tried to clarify [94]. Among other challenges and limitations these new tools hide, we can mention the risk of sample and response bias. For instance, despite the great potential for recruitment in rural/suburban areas, online surveys restrict the selection to those interested in the topic and may exclude lower socioeconomic groups. Similarly, it is also difficult to prove the reliability of declared data [95,96].

With particular reference to the neuropsychological effects of COVID-19 in children, this is one of the few studies including a COVID-19-negative control group. This is to better investigate the psychosocial influence of the pandemic and compare it with the direct role of SARS-CoV-2 on infected patients.

While previous studies have used the EQ-5D-Y to assess HRQoL in pediatric populations with specific chronic conditions, our study stands out as the first to employ this tool for evaluating HRQoL in children and adolescents during the SARS-CoV-2 pandemic [97].

However, the lack of a prospective design did not allow for the evaluation of symptoms’ evolution, especially in Long COVID-19 patients. In addition, we could not determine the temporal relation between SARS-CoV-2 infection and vaccination due to a lack of data on vaccination times.

Therefore, although we cannot draw general conclusions on the long-term impact of COVID-19, our results confirmed the negative effect on social habits and mental well-being in the general pediatric population and, in particular, on a subgroup of patients probably susceptible to prolonged neuropsychological symptoms after acute infection.

## 5. Conclusions

Our results underline that SARS-CoV-2 has spared no age group of the pediatric population, regardless of the infection contractions. Indeed, if, on one side, uninfected children more frequently reported changes in sleep, appetite, and PA and an increased need for medical/psychological support, on the other, the subgroup of patients reporting persistent symptoms after acute COVID-19 resulted in being more susceptible to emotional instability and fatigue.

The relatively low impairment of social habits in the general COVID group may be explained by the common mild course of acute infection in pediatric age and confirmed by QoL scores comparable to controls. On the contrary, the Long COVID group seems to be more susceptible to QoL deterioration. Of note, these patients also showed a certain level of pre-existing mood impairment, a result quite unexpected that deserves further investigation.

The perception of psychological problems among non-infected subjects and the persistence of neuropsychological impairment in the Long COVID-19 group should encourage the monitoring of these phenomena. For this purpose, new digital technologies and telemedicine are fundamental for the screening and early identification of neuropsychiatric risk factors. Preventive and intervention strategies of social welfare involving the whole family, together with a sensible enhancement of pediatric mental-health care services, are pivotal to facing this challenge of the present day.

## Figures and Tables

**Table 1:** Questions related to social relationships and emotional well-being.

**Compared to before your illness/pandemic onset, how much are you now doing/experiencing the following:**
Eating
Sleeping
Physical activity
Fatigue
Spending time with friends in person
Spending time with friends remotely
Spending time watching TV, playing video/computer games, or using social media for educational purposes, including school/nursery work
Spending time watching TV, playing video/computer games, or using social media for non-educational purposes
Spending time outside
Attending nursery/school/university/work
**Compared to before your illness/pandemic onset: Have there been changes in your…**
Connections with others
Emotions
**Compared to before your illness/pandemic onset: Have there been changes in your…**
Behavior
Relationships, in how they get on with others

**Table 2:** Questions related to quality of life from EuroQoL EQ-5D-Y-3L version. (*) The answer included three possible choices: no problems (level 1), some problems (level 2), or a lot of problems (level 3).

Under each heading, please tick the ONE box that describes your health TODAY *
Mobility (walking about)
Looking after myself
Doing my usual activities
Having pain or discomfort
Feeling worried, sad, or unhappy
Under each heading, please tick the ONE box that describes your health BEFORE the onset of your illness/pandemic outbreak *
Mobility (walking about)
Looking after myself
Doing my usual activities
Having pain or discomfort
Feeling worried, sad, or unhappy

**Table 3:** Demographic characteristics of the population included in the analysis by COVID-19 status.

	COVID-19 n = 396	Controls n = 111	*p*-Value
**Gender, n (%):**			0.316
Male	194 (49.0)	61 (55.0)	
Female	202 (51.0)	50 (45.0)	
**Ethnicity, n(%):**			0.129
Caucasian	253 (64.1)	75 (67.6)	
Other	48 (12.0)	7 (6.3)	
Not specified	95 (23.9)	29 (26.1)	
**Age, years**	9.32 ± 2.55	9.41 ± 2.74	0.747
**Class of Age, n (%):**			0.224
6–12 years old	348 (87.9)	92 (82.9)	
13–18 years old	48 (12.1)	19 (17.1)	
**Vaccination** **, n (%):**			0.003
Yes	138 (55.9)	81 (72.9)	
No	109 (44.1)	30 (27.1)	

## Data Availability

Data available on request due to restrictions. The data presented in this study are available on request from the corresponding author. The data are not publicly available due to privacy.
